# Identification of an exosite at the neutrophil elastase/alpha‐1‐antitrypsin interface

**DOI:** 10.1111/febs.17387

**Published:** 2025-01-08

**Authors:** Roberto Gangemi, Mattia Bignotti, Andrea Denardo, Claudia N. Pearce, Riccardo Ronzoni, David A. Lomas, James A. Irving, Annamaria Fra, Fabrizio Gangemi

**Affiliations:** ^1^ Physics, Department of Molecular and Translational Medicine University of Brescia Italy; ^2^ Experimental Oncology and Immunology, Department of Molecular and Translational Medicine University of Brescia Italy; ^3^ UCL Respiratory and the Institute of Structural and Molecular Biology University College London UK

**Keywords:** alpha‐1‐antitrypsin deficiency, computational biology, molecular dynamics simulations, protease inhibition, protein–protein interactions, SERPINA1, Serpins

## Abstract

Neutrophil elastase (NE) is released by activated neutrophils during an inflammatory response and exerts proteolytic activity on elastin and other extracellular matrix components. This protease is rapidly inhibited by the plasma serine protease inhibitor alpha‐1‐antitrypsin (AAT), and the importance of this protective activity on lung tissue is highlighted by the development of early onset emphysema in individuals with AAT deficiency. As a serpin, AAT presents a surface‐exposed reactive centre loop (RCL) whose sequence mirrors the target protease specificity. Following binding of NE in a ‘Michaelis’ encounter complex, cleavage of the RCL results in an irreversible complex between the two molecules. Here, the structure of the AAT‐NE encounter complex was studied by molecular dynamics, mutagenesis and enzyme kinetics. Exploration of the geometry of interaction between the two molecules revealed the possibility that the interaction interface extends beyond the RCL; a persistent feature of the simulations was the interaction between a region located upstream of β‐strand 4C of AAT, comprising three acidic residues (Asp202, Glu199 and Glu204), and Arg147 of NE. Mutation of the acidic residues to either alanine or serine, or a D202R substitution, resulted in a reduced rate of association between recombinant AAT and NE. Addition of salt to the buffer had little effect for these mutants but substantially reduced the rate of interaction of the wild‐type protein. These data are consistent with a role for this acidic region on AAT as an exosite that contributes to an optimal interaction with its physiological protease target.

## Graphical abstract

Molecular dynamics simulations predicted an exosite in the encounter complex between alpha‐1 antitrypsin (AAT) and neutrophil elastase (NE), comprising a patch of acidic residues which interacts with a basic loop on NE. Recombinant AAT variants with mutations of the three acidic residues show reduced association rate compared to wild‐type and confirm the role of the exosite in the initial AAT‐NE interaction.

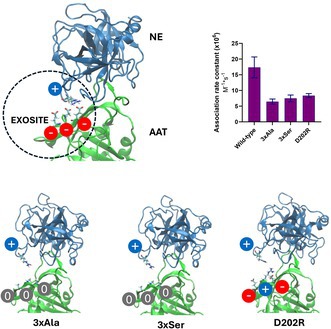

**Abbreviations**
AAT: alpha‐1‐antitrypsin
AATD: alpha‐1‐antitrypsin deficiency
CD: circular dichroism
ED: essential dynamics
IHB: intermolecular hydrogen bond
MD: molecular dynamics
MMGB/SA: molecular mechanics generalised Born/surface area method
NE: neutrophil elastase
RCL: reactive centre loop
RMSD: root mean square deviation
RMSF: root mean square fluctuation
SASA: solvent accessible surface area
SI: stoichiometry of inhibition

## Introduction

Alpha‐1‐antitrypsin (AAT) is an abundant plasma protein belonging to the serpin family of protease inhibitors. Constitutively released by hepatocytes and upregulated during the acute phase of an inflammatory response, AAT inhibits chymotrypsin‐like serine proteases released during degranulation of neutrophils; the most rapid and efficient interaction occurs with neutrophil elastase (NE), with some activity against proteinase‐3 [1, 2]. The inherited disorder alpha‐1‐antitrypsin deficiency (AATD) highlights the role of AAT in regulating protease activity during innate inflammatory responses. In severe states of AATD, such as those occurring in homozygote carriers of the Z‐AAT mutant (E342K), plasma levels of AAT diminish below a protective threshold leading to uncontrolled activity of the elastolytic neutrophil proteases on the lung tissue, progressive damage of the alveolar walls and elevated risk of early onset emphysema [3, 4].

**Fig. 1:**
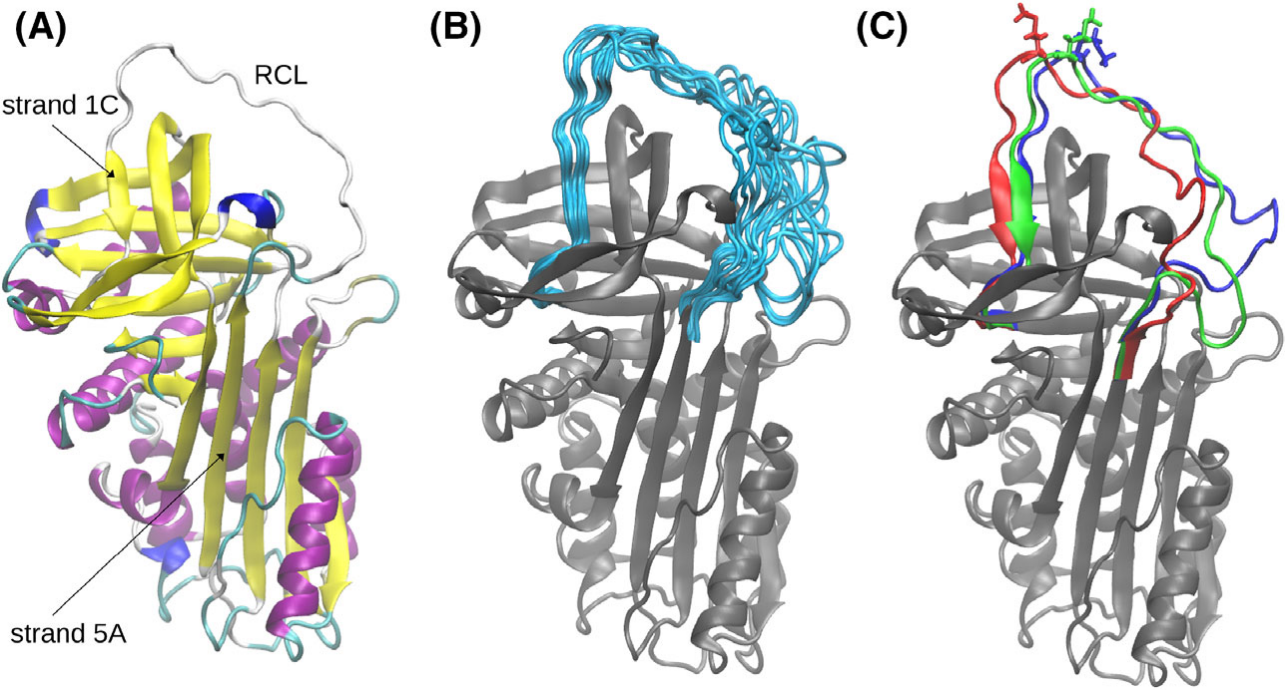
Dynamics of the RCL. (A) AAT structure from PDB 1QLP. Colours refer to secondary structure: yellow for β‐sheet, purple for α‐helix, blue for 3–10 helix, cyan for turn, white for coil. (B) Twenty representative RCL conformations obtained by use of the *k*‐means algorithm superimposed onto the 1QLP structure of AAT (grey). (C) The three structures derived from cluster analysis that were used for the subsequent simulations, with residue P1 (Met358) highlighted as sticks. Structural representations created by means of VMD [41].

AAT has a typical serpin fold, comprised of three β‐sheets surrounded by a network of nine α‐helices. A key structural element for protease inhibition by serpins is the reactive centre loop (RCL), an exposed loop that mediates serpin‐protease specificity by acting as a ‘bait’ region and whose structural configuration is optimal for engagement with the protease catalytic site (Fig. 1A) [5].

Inhibition proceeds via a ‘suicide‐substrate’ mechanism [6], initiated when the protease forms a noncovalent encounter (Michaelis) complex, dictated by conformational, steric and physicochemical complementarity of its active site cleft for the serpin RCL. This brings the AAT pseudo‐substrate peptidyl bond, located between residue positions denoted P1 and P1′ [6, 7, 8], into an appropriate geometry with the protease catalytic His, Asp and Ser residues. The canonical mechanism of hydrolysis of a substrate by a chymotrypsin‐like serine protease involves deprotonation of the Ser195 side chain by His57, whose orientation is stabilised by Asp102. The resulting reactive Oγ atom of Ser195 then exerts a nucleophilic attack on the carbonyl C‐atom of the P1 residue of the substrate, forming a covalent acyl‐protease intermediate linking the two molecules. The concomitant disruption of the P1‐P1′ peptide bond allows the serpin to undergo a conformational transition whereby the proximal segment of the RCL—covalently bound to the protease—inserts into the centre of the serpin's β‐sheet A. Distinct phases of insertion have been observed in kinetic experiments, with an initial rapid step around mid‐way down β‐sheet A, followed by a slower phase associated with partial remodelling of an occluding helix [9]. In the process, the protease is translocated to the opposite side of the serpin, and in the final complex, its active site is distorted and unable to hydrolyse the ester bond connecting the two molecules [10]. This requires timely and coordinated changes within the serpin; interference with the dynamics of insertion of the RCL—for example, as a result of dysfunctional RCL mutations [11, 12]—can lead to de‐acylation and subsequent dissociation of the complex, whereby the protease returns to its native active structure, and the cleaved serpin remains inactive.

In some serpins, exosite contacts—those outside this primary region of binding—have been found to contribute to target protease specificity. A notable example is the role of an exosite in heparin‐induced conformational change of antithrombin that increases its rate of inhibition of coagulation factors IXa and Xa [13]. Such contacts can modulate the relative specificity of serpins with respect to different proteases by extending the interface in the encounter complex [14, 15].

In the case of AAT, no exosite has been identified to date. However, there is circumstantial evidence that supports the contribution of factors extrinsic to the RCL alone in the inhibition of the physiological target, neutrophil elastase (NE). It has been observed that AAT exhibits a high rate of inhibition of this latter protease at odds with a much lower kinetic constant against peptide substrates with the RCL sequence [1, 16]. Furthermore, transplantation of this region onto the antichymotrypsin scaffold yielded a chimeric protein that showed a poor rate of association with NE. In considering other chimeric mutants, the authors concluded that one of the contributing factors was the presence of an unidentified secondary‐binding site [17]. Similarly, a serpin scaffold engineered using consensus design principles with the RCL of AAT, conserpin, showed a lower rate of association with NE than the full AAT protein [18].

Despite the importance of the interaction between AAT and NE in maintenance of tissue integrity during an inflammatory response, there is no available crystallographic or cryo‐EM structure of a complex between these molecules. Some experimental evidence for the likely relative orientation of the reactants is found in the X‐ray structure (PDB 1OPH) of the complex between the Pittsburgh (M358R) variant of AAT and variant S195A of bovine trypsin [19]. However, as trypsin and NE share only around 30% identity, this structure cannot encapsulate the detailed interactions occurring between the wild‐type RCL of AAT and its physiologically cognate protease NE. Indeed, in this paper, the authors observed that intermolecular contacts with trypsin were more limited than expected. It is well‐established that crystal structures are by definition subject to constraints imposed by contacts in the crystal lattice and as such they represent ‘snapshots’, whereas other computational (molecular dynamics) and experimental (NMR) techniques are required to extract information on the conformational dynamics of a system.

Accordingly, here we have performed a detailed molecular dynamics and biochemical investigation of the interaction between NE and AAT. To achieve this, we have generated a starting model of this physiological pair based on the above‐mentioned crystal structure. The analysis of the structural properties of the interface and of the key contacts identified an electrostatic exosite interaction between a positively charged loop of NE and a negatively charged segment preceding β‐strand 4C of AAT. The relevance of this interface to the formation of the resulting complex between these molecules was confirmed experimentally using site‐directed mutagenesis and kinetic assays.

## Results

The computational strategy adopted in this work to generate and evaluate a detailed model of the AAT‐NE Michaelis complex proceeded as follows: (a) analysis of the RCL structure and dynamics identified the most significant conformations compatible with the interaction with NE; (b) a model of the complex was created based on the AAT Pittsburgh‐trypsin crystal, and a hybrid molecular dynamics (MD)/essential dynamics (ED) approach was used to induce complex formation and equilibrate the structure; (c) the catalytic site geometry allowed assessment of the resulting structure, and standard MD simulations were performed to establish the stability of the complex and to achieve an accurate sampling of the conformational space for subsequent structural analyses.

### Structure and dynamics of the RCL of alpha‐1‐antitrypsin

As molecules in a protein crystal are subject to steric constraints and contacts imposed by the crystal lattice, the resulting structures lack information on large‐scale molecular motions and may not entirely recapitulate the preferred state in solution. Accordingly, as a basis for modelling of the docking complex with NE, the X‐ray crystal structure of native, uncomplexed AAT (PDB 1QLP) [5] (Fig. 1A) was subjected to four independent 500 ns MD simulations in explicit solvent to determine relevant conformational sub‐states of the RCL compatible with complex formation. The four trajectories were merged into a single sample of 2 μs (with one frame every 40 ps), and the structures at each timepoint were aligned to a common reference to remove overall translations and rotations. The subsequent structural and dynamical analysis was restricted to the backbone and Cβ atoms of the RCL and adjacent strands 5A and 1C (residues 339 to 371); these latter elements displayed low root mean square fluctuations (RMSF) and could be considered as fixed with respect to the rest of the protein.

To identify discrete RCL conformations, the *k‐means* algorithm was applied to divide the sample into 20 clusters and the average RCL coordinates of each cluster determined. A comparison of these (Fig. 1B) revealed a large variability in residues 343 to 355, with more stability in the C‐terminal part of the RCL. For each cluster, a single representative frame was selected from the original trajectory that most closely matched within the RCL region. Among the twenty AAT models thus obtained, three had a side chain orientation of the P1 residue (Met358) compatible with NE binding (Fig. 1C) and were progressed for further analysis.

### Formation of the Michaelis AAT‐NE docking complex

The X‐ray crystal structure of the complex between the AAT Pittsburgh variant (M358R) and the inactive S195A mutant of bovine trypsin (PDB 1OPH) [19] was used as a reference for a suitably oriented pre‐encounter complex for subsequent MD simulations. Each of the three AAT models of RCL conformation shown in Fig. 1C was superposed against the AAT Pittsburgh component of the 1OPH structure, while the crystal structure of human NE (PDB 3Q76) [20] was superimposed on bovine trypsin by means of an overall best fit. The three structures thus obtained were then perturbed by increasing the distance between AAT and NE along the vector connecting their centres of mass by 7 Å, a distance found to minimise clashes but allow complex formation within a reasonable timeframe.

**Fig. 2:**
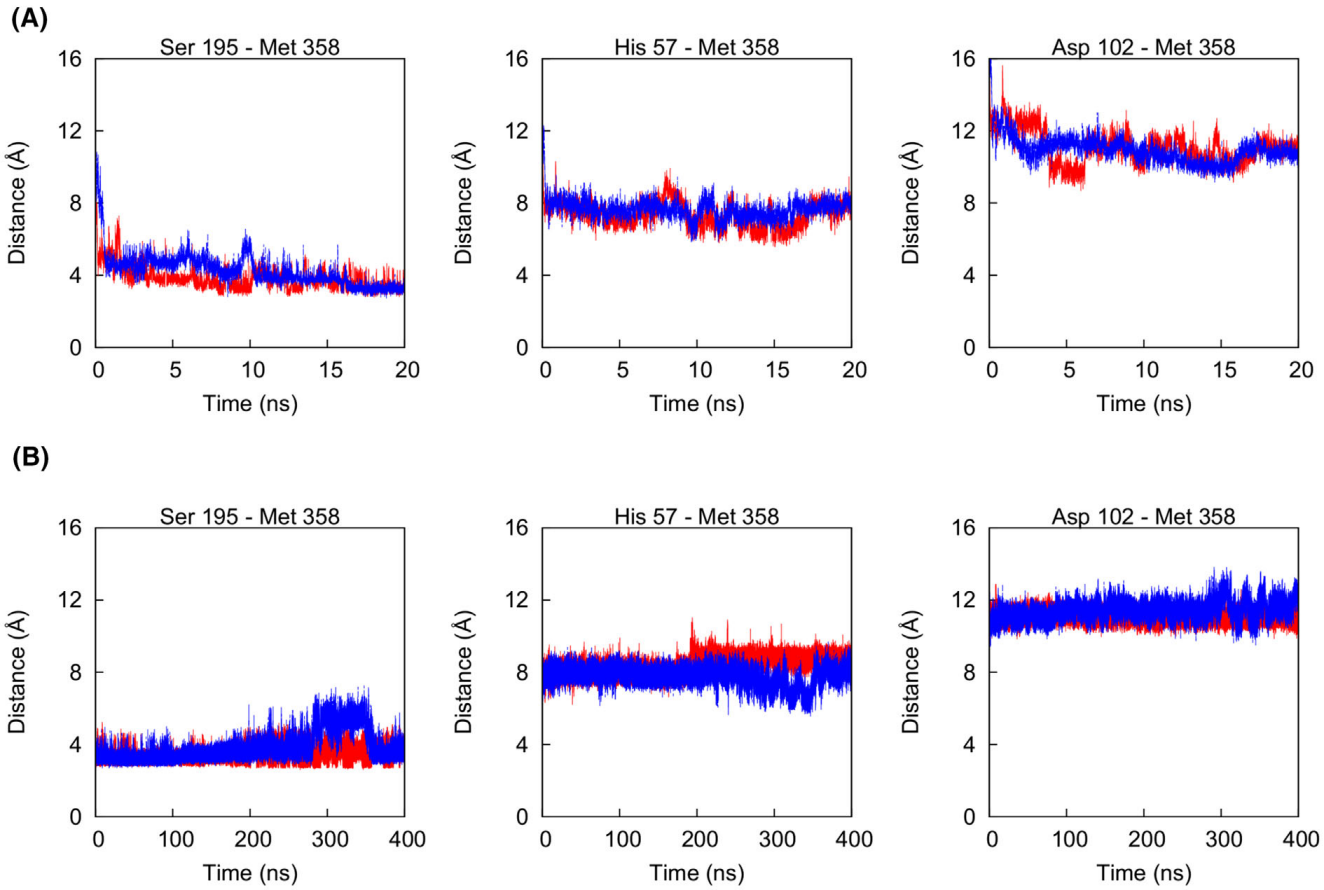
Progress of the ED and standard MD simulations. (A) Time evolution of the distances of reference atoms in the catalytic triad of NE with respect to Met358 of AAT in two ED simulations (in red and blue) that produced an acceptable Michaelis complex. (B) The same as A in the two subsequent standard MD simulations. The reference atoms are Oγ for Ser195 and carbonyl C for Met358 in the left panel, and Cα for each residue in the central and right panels.

Each of the three models was then subjected to 10 independent essential dynamics (ED) simulations of 10 ns length, in which the collective variable used was the distance between the centres of mass of AAT and NE. The steered dynamics algorithm applied caused this collective variable to progressively decrease by periodically rejecting moves in which its value increased. Importantly, this approach does not alter the inherent dynamics of a system while allowing it to evolve in the desired direction in a computationally feasible amount of time. In two of the thirty simulations, the reactive atom Oγ of NE Ser195 and the target carbonyl C‐atom of the AAT P1 Met358 became steadily lower than 4 Å after 10 ns, a precursor step to nucleophilic attack on the RCL at the P1‐P1′ peptide bond. These two ED simulations were extended to 20 ns to allow optimisation of the contacts at the interface. The time evolution of the distances of Met358 from the catalytic triad (Ser195, Asp102, His57) for one trajectory is shown in Fig. 2A, and the initial part of one ED simulation is reported in Videos S1 and S2.

**Fig. 3:**
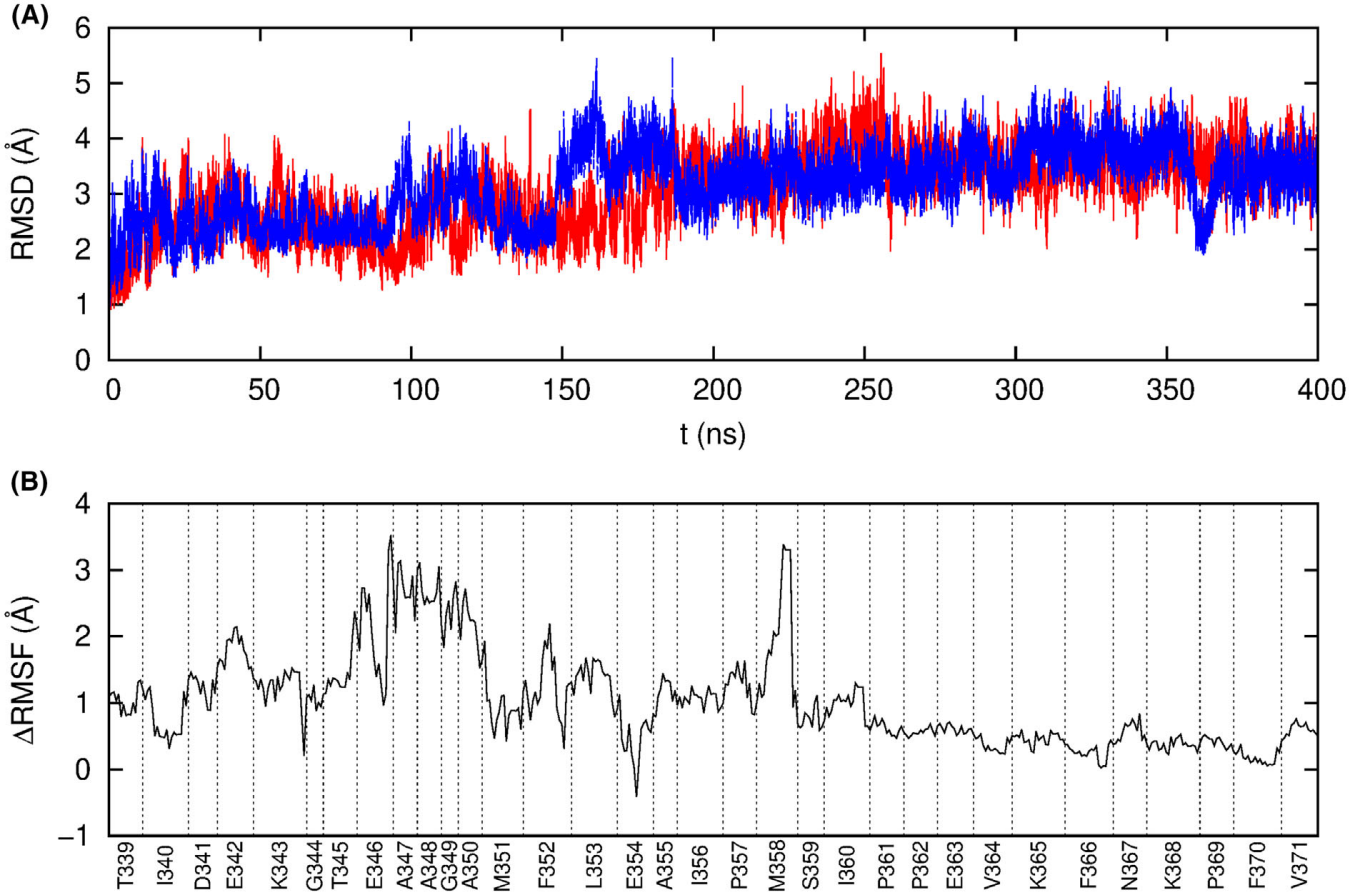
Analysis of standard MD simulations. (A) Time evolution of the root mean square deviation of Ca atoms with respect to the initial structure in two standard MD simulations (in red and blue). Subsequent analyses used a combination of the last 200 ns of both trajectories. (B) Analysis of the difference in atomic root mean square fluctuation (RMSF) of residues 339–371, encompassing the RCL and adjacent positions, between AAT alone and in complex with NE.

The two models obtained by essential dynamics were then subjected to equilibration by means of 400 ns standard MD simulations. The time evolution of the root mean square deviation (RMSD) of the Cα atoms revealed that after approximately 200 ns the structures had stabilised (Fig. 3A); therefore, the subsequent analyses were based on the last 200 ns of both trajectories, giving a combined sampling time of 400 ns.

The overall dynamics of the AAT‐NE complex was analysed by means of principal component analysis, based on the backbone, Cα, and Cβ atoms. The three most important components (accounting for 70% of the total variance) involved essentially rigid rotations of NE around three almost orthogonal axes, while AAT underwent rotations combined with deformations of various regions, including the RCL. These motions are shown in three Figures S1–S3. The persistence of large RMSD fluctuations throughout the MD trajectories was mainly related to such motions of the whole complex.

Examination of the root mean square fluctuation (RMSF) of residues 339–371 in the AAT‐NE complex and in the uncomplexed AAT showed RCL mobility to be reduced in the former case (Fig. 3B). The regions that were more constrained by the presence of NE appeared to be around residues 347–350, 358 and 360. Other parts of the RCL, especially the C‐terminal end, were essentially unaffected by the presence of the bound protease. This is compatible with a hypothesis that, when unbound, the RCL retains a ‘substrate‐like’ conformation [19, 21]. However, when considering the contacts within the interface region, especially those involved in the catalytic reaction, a higher stability was observed (Fig. 2B).

### The AAT‐NE complex shows engagement between Met358 and the catalytic triad consistent with the Michaelis complex

**Fig. 4:**
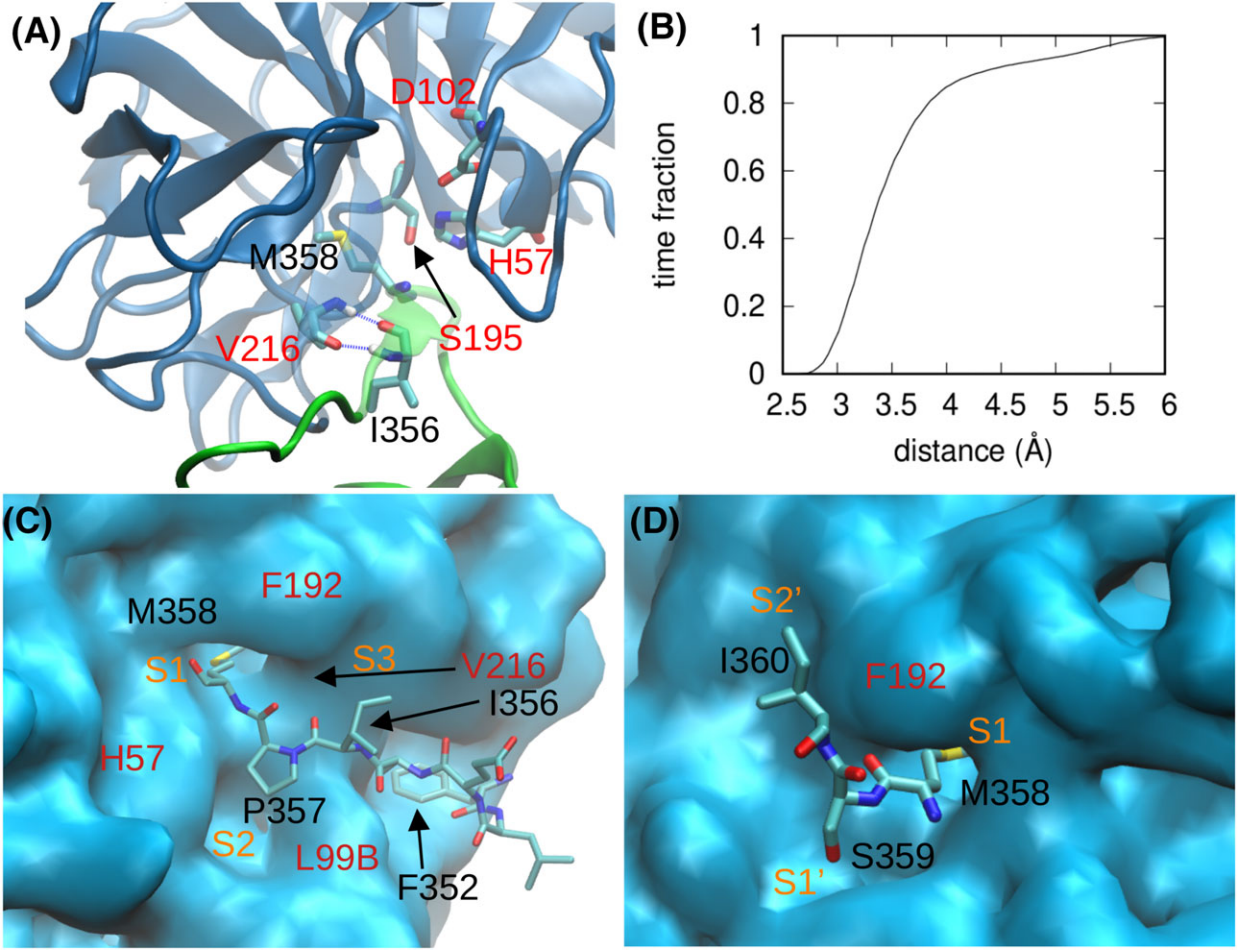
Structure of the AAT‐NE complex from standard MD. (A) Detail of the active site, with the main structure in cartoon representation (green for AAT, blue for NE) and relevant residues highlighted as sticks. Residue labels are in black for AAT and in red for NE. The double hydrogen bond between Ile356 and Val216 is also shown. (B) Fraction of the time in which the distance between Oγ of Ser195 on NE and the carbonyl of Met358 on AAT is lower than the value given in abscissa. (C) Position of the portion of the RCL from P1 to P7 (shown as sticks) with respect to NE (shown as surface). Label colours are as in A, and subsite labels are shown in orange. (D) The same as C for the part of RCL including P1, P1′ and P2′. Structural representations created by means of VMD [41].

Inspection of the interface region maintained in the simulations revealed a geometry that is compatible with the formation of a reaction intermediate. With some fluctuation, on average the residues of the catalytic triad adopted the correct position with Ser195 oriented towards the RCL backbone in position P1, and the His57 side chain between those of Ser195 and Asp102. A typical MD snapshot is shown in Fig. 4A, in which the distance of the atom Oγ of Ser195 on NE from the carbonyl of Met358 on AAT showed an average value of 3.3 Å, and around 10% of the time it was in the range 2.65–3 Å (Fig. 4B). These values compare favourably with a reaction intermediate corresponding to a Michaelis complex (PDB 1TAW) with a distance of 2.73 Å [22]. The compatibility of the state described by MD trajectories with an AAT‐NE Michaelis complex is further supported by the analysis (Fig. 4C,D) of subsites of NE [6, 7]: the most relevant RCL residues around P1 (Met358), in particular P2 (Pro357), P3 (Ile356), P1′ (Ser359) and P2′ (Ile360) occupy the expected pockets.

### The AAT‐NE interface includes extensive contacts

**Fig. 5:**
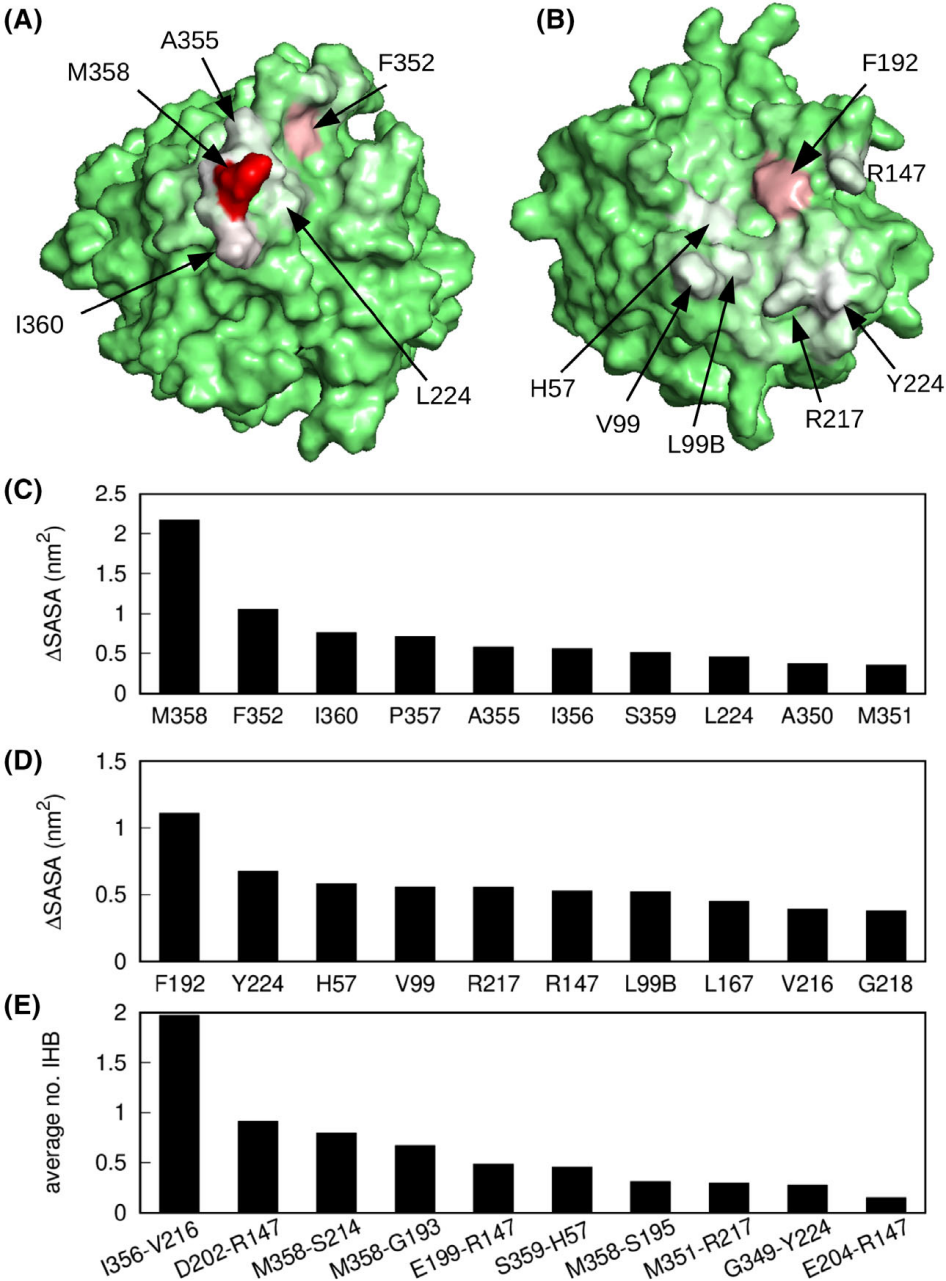
Analysis of AAT‐NE interactions. (A) Colour representation of SASA loss of AAT residues upon binding (green: no variation; white: 1.1 nm^2^; red: 2.2 nm^2^). (B) Colour representation of SASA loss of NE residues upon binding (colours as in A). (C) Variation of SASA upon binding: the 10 largest values among AAT residues are shown. (D) Variation of SASA upon binding: 10 largest values among NE residues. (E) AAT‐NE residue pairs with the highest average number of intermolecular hydrogen bonds (IHB). Structural representations created by means of VMD [41].

The extent of the broader interface between the protease and inhibitor was considered by identifying residues with reduced solvent accessibility in the complex with respect to the uncomplexed molecules. The average difference between solvent accessible surface area (SASA) for each amino acid was estimated across the trajectory and is shown by colour codes in Fig. 5A,B; the 10 residues from each structure with the largest loss of SASA are shown in Fig. 5C,D. The majority of these report hydrophobic contacts, and notably, all but one of the AAT residues arise from the RCL, most substantially the P1 Met358 due to its occupation of the narrow hydrophobic S1 subsite of NE.

The persistence of intermolecular hydrogen bonds (IHB) was also determined and the 10 highest values are shown in Fig. 5E. A double IHB involving the backbone atoms of Ile356 on AAT and Val216 on NE was particularly stable, present for about 99% of the simulation time and also found in the trypsin‐AAT crystal structure, creating an edge‐β‐strand interaction (see Fig. 4A). Backbone atoms of Met358 variously formed bonds with NE residues Gly193, Ser214 or Ser195, engaging in 2 IHBs in 89% of the simulation time. This, in addition to the above‐mentioned hydrophobic interactions, highlights the crucial role of this region of the RCL in the formation of the AAT‐NE Michaelis complex.

### The MD simulations reveal the presence of an exosite that mediates an intermolecular interaction

**Fig. 6:**
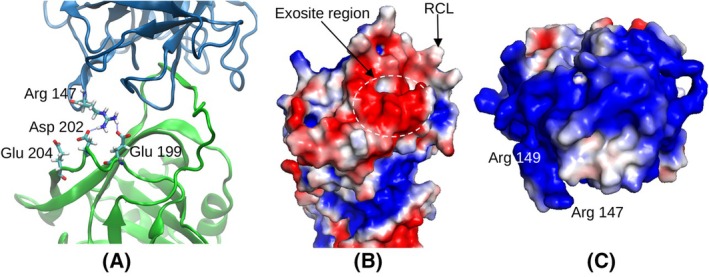
Analysis of the putative exosite region. (A) Details of the exosite interactions, where the involved amino acids are highlighted as sticks and the main structure is in cartoon representation (green for AAT, blue for NE). (B) Surface electrostatic potential of AAT shown by colour coding (red: –2kT/e, blue: +2kT/e). (C) Surface electrostatic potential of NE (colours as in B). Structural representations created by means of VMD [41] (A) and Pymol (Schrödinger, LLC) (B and C).

Outside the RCL, it was observed that negatively charged AAT residues Asp202, Glu199 and Glu204 showed a favourable and relatively consistent interaction with NE residue Arg147 with an average number of IHBs of 1.55 per frame over the course of the simulations (Fig. 5E), forming at least 1 IHB for 70% of time, at least 2 IHBs for 59% of time and at least 3 IHBs for 22% of time. A typical configuration of these hydrogen bonds is shown in Fig. 6A.

While previous studies have considered the role of the RCL in the AAT‐NE interaction, a secondary exosite has not been described previously. Analysis of the distribution of the electrostatic potential at the surfaces of NE and of AAT clearly shows a negatively charged region involving the three acidic residues of AAT (Fig. 6B) and a positively charged region around Arg147 of NE that includes the adjacent Arg 149 (Fig. 6C).

**Fig. 7:**
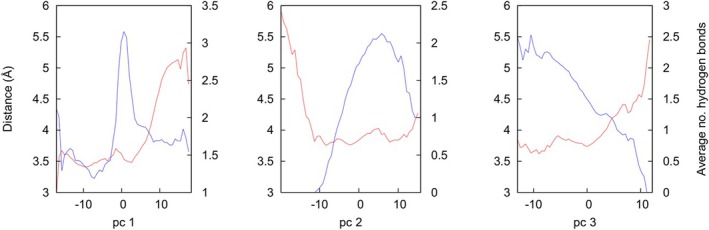
Correlation between exosite interaction and catalytic site geometry. Distance between the carbonyl of Met358 and atom Oγ of Ser195 (red curves, left scale) and average number of hydrogen bonds between Arg147 and AAT (blue curves, right scale) as a function of the first (left panel), second (central panel) and third (right panel) principal component of the AAT‐NE complex, calculated from the two MD simulations as described in the text.

To assess the potential role of the exosite in coordinating the interaction between the two molecules, the correspondence to a key metric of the inhibitory mechanism—the distance between the carbonyl C‐atom of Met358 and the Oγ atom of Ser195, d_CO_—was assessed. This distance is affected by the motions of the complex, which can be decomposed to orthogonal movements by means of the principal components discussed above. For each of the three principal components describing the largest movements, we divided the range of observed values of the corresponding collective coordinate into 50 bins and compared the average value of d_CO_ in each bin to the average number of hydrogen bonds mediated by NE Arg147 (Fig. 7). For the second and third principal components (central and right panels of Fig. 7), broadly reflecting transverse tilting of NE with respect to the central segment of the RCL and torsion around the vertical axis respectively (see Figures S2 and S3), increased hydrogen bonding in this region corresponded with P1‐active site proximity (low d_CO_), while this was partially the case for the first principal component (Fig. 7, left panel) consisting of longitudinal tilting of NE with respect to the central segment of the RCL (Figure S1).

These observations collectively suggest that the putative exosite may help to coordinate an appropriate alignment between the two molecules.

### Perturbation of the putative exosite reduces the rate of association by NE

The existence of the possible exosite was investigated experimentally by mutagenesis of residues Glu199, Asp202 and Glu204 and evaluation of the effects on inhibition of NE. Three mutants of AAT were considered: one in which the residues were replaced by alanine so as to remove any charged or polar group (3×Ala); one where the residues were replaced by serine, thus removing their net negative charge, but retaining polar side chains (3×Ser); and a point mutant in which Asp202 was replaced by arginine, thus reversing the charge of the central residue of the acidic patch that was most important according to the MD results (D202R).

**Fig. 8:**
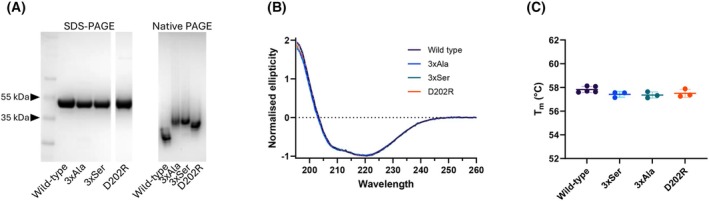
Amino acid substitutions within the putative exosite do not affect protein conformation. (A) Recombinant AAT variants analysed either by 4–12% w/v acrylamide SDS/PAGE in reducing conditions (left) or by 4–12% w/v acrylamide nondenaturing‐PAGE (right). Proteins were stained by 0.1% w/v Coomassie Blue. (B) Circular dichroism spectrum of wild‐type and AAT mutants recorded at 25 °C; the mean of *n* = 3 dilutions is shown. (C) Thermal transition midpoints (*T*_*m*_) determined in differential scanning fluorimetry experiments using SYPRO Orange as a reporter and an increment of 1 °C·min^−1^; individual values (*n* = 3–5) are shown with mean ± SD.

These mutants were expressed in *E. coli*, purified by affinity chromatography, and were confirmed to be in a pure monomeric form by SDS and nondenaturing PAGE (Fig. 8A). Circular dichroism (CD) (Fig. 8B) and thermal stability (Fig. 8C) analyses confirmed that these substitutions did not detectably perturb their overall structure. Consistently, no significant distortions were found when comparing, by RMSD of Cα, the average structures from 200 ns MD simulations of the three AAT variants with the average from the 2 μs aggregate wild‐type trajectory: the values of 1.44 Å, 1.56 Å, and 1.28 Å for 3×Ala, 3×Ser, and D202R, respectively, are very similar to those obtained for each wild‐type simulation, in the range of 1–2 Å.

**Fig. 9:**
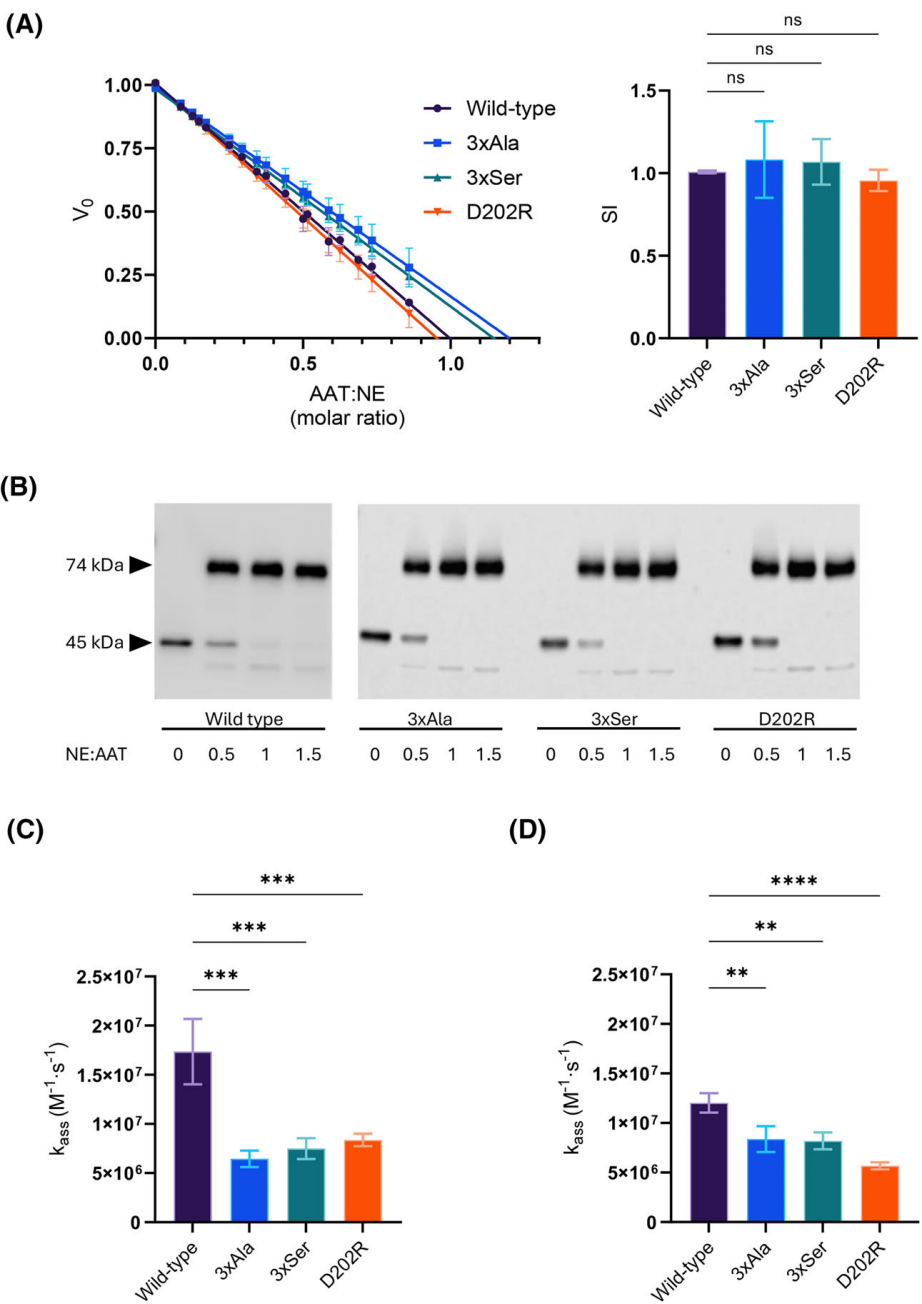
Amino acid substitutions at the exosite decrease the rate of association by NE. (A) Stoichiometry of inhibition of NE by recombinant AAT mutants compared to wild‐type AAT. The rate of hydrolysis (V_0_) of a chromogenic substrate was determined at different AAT : NE molar ratios and fitted by linear regression. The histogram shows the SI values, defined as the number of AAT molecules required to inhibit a molecule of NE, calculated as the means of the x‐intercepts (*n* = 3). Error bars represent ±SD values. (B) Analysis of covalent complex formation by SDS/PAGE, at the indicated NE : AAT ratios. (C) Second‐order association rate constant (*k*_ass_), corrected for nonproductive interactions, was calculated as the product of *k*_inh_ and the SI mean (see Material and Methods). The association rate was determined in PBS. (D) Second‐order association rate constant (*k*_ass_) determined as in panel C, in PBS with 0.5 m NaCl (*n* = 3). Data in panels A, C and D are represented as means ± SD (*n* = 3). Significant differences from wild‐type AAT were calculated by one‐way ANOVA analysis corrected by Dunnett's test (ns, nonsignificant; **, *P* ≤ 0.01; ***, *P* ≤ 0.001; ****, *P* ≤ 0.0001), using Prism 10 (graphpad software).

The stoichiometry of inhibition (SI), representing the number of AAT molecules required to inhibit one molecule of NE, is a measure of the efficiency of the inhibitory mechanism when a productive interaction occurs. Wild‐type AAT has an SI of 1, reflecting the timely incorporation of the RCL into β‐sheet A and the proper adoption of the fully inserted conformation. In an end‐point kinetic experiment evaluating the residual protease activity at different relative concentrations of AAT, the SI values of the AAT mutants were found to be not significantly different from that of wild‐type AAT (Table 1, Fig. 9A). This suggested minimal impacts of the mutations on the integrity of the molecule and the proper functioning of the inhibitory mechanism and was confirmed by SDS/PAGE which showed formation of covalent AAT‐NE complexes (Fig. 9B).

**Table 1:** Stoichiometry of inhibition (SI) and second‐order association rate constants corrected for nonproductive AAT turnover (*k*_ass_). The association rate was determined in two buffers with different NaCl concentration. Data are reported as mean ± SD (*n* = 3).

	SI ± SD	*k*_ass_ (m ^−1^ s^−1^) ± SD	*k*_ass_ (m ^−1^ s^−1^) ± SD
		PBS	PBS + 0.5 m NaCl
Wild‐type	1.00 ± 0.01	17.4·10^6^ ± 3.3·10^6^	11.8·10^6^ ± 1.0·10^6^
3×Ala	1.20 ± 0.23	6.5·10^6^ ± 0.8·10^6^	8.4·10^6^ ± 1.3·10^6^
3×Ser	1.15 ± 0.14	7.5·10^6^ ± 1.1·10^6^	8.2·10^6^ ± 0.9·10^6^
D202R	0.93 ± 0.06	8.3·10^6^ ± 0.6·10^6^	5.7·10^6^ ± 0.4·10^6^

The second‐order rate constant of inhibition reports instead the rate of first formation of the docking complex between the two molecules. The time‐dependent inhibition of NE by the mutants was followed using the progress curve method and the association rate constant determined for each (Table 1, Fig. 9C). Strikingly, in a PBS buffer there was a clear reduction in the rate of interaction in the absence of the three charged residues, and when an unfavourable repulsive charge was introduced by the D202R mutation (Fig. 9C). Addition of a further 0.5 m NaCl to the buffer substantially decreased the rate of interaction between wild‐type AAT and NE with some effect on D202R and little effect on the triple‐mutants (Fig. 9D). This is consistent with shielding of an electrostatic interaction between the two molecules and that this interaction is mediated by the region identified in AAT. Together these results are consistent with a mechanism in which the Glu199/Asp202/Glu204 site provides a favourable interaction that is important for optimal binding of the protease to AAT, supporting the inference of an exosite drawn from the MD simulations.

### MD simulations of mutants support the role of the putative exosite

**Fig. 10:**
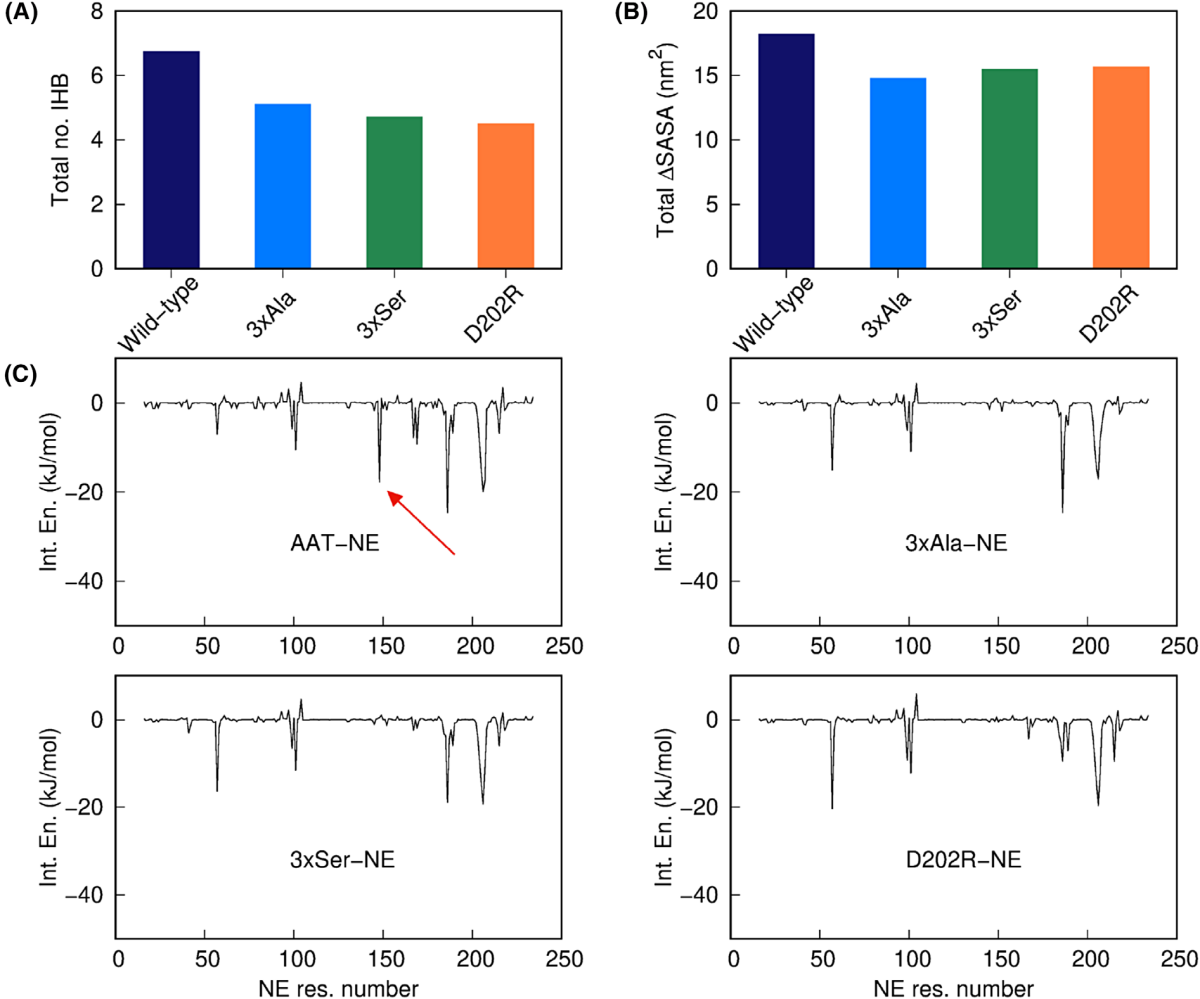
Comparison of MD results between wild‐type and mutant complexes. (A) Total number of intermolecular hydrogen bonds between AAT and NE. (B) Total loss of SASA of AAT and NE upon complex formation. (C) Contributions by NE residues to the interaction energy with AAT (a red arrow in the wild‐type case points to the peak of Arg147, interacting with the putative exosite).

To place these *in vitro* results in the context of the computational characterisations, we performed 400 ns simulations of the three mutant AAT‐NE complexes, with starting structures obtained by applying mutations to a snapshot from MD simulations of the wild‐type complex. IHBs and loss of solvent accessible surface area, averaged over the last 200 ns of each trajectory, clearly show a reduction of both polar and hydrophobic interactions in all mutants relative to wild‐type (Fig. 10A,B). Estimation of the interaction energies by the MMGB/SA [23, 24] method, which has the advantage of providing a breakdown of single‐residue contributions to the total energy, showed a clear contribution of Arg147 in the wild‐type but absent in the complexes formed by the mutants (Fig. 10C). These results further support the role of Arg147 in the binding of NE with AAT.

## Discussion

Serpins are crucial regulators of proteases in physiologically important pathways, such as blood coagulation, fibrinolysis and inflammation. The serine and cysteine proteases regulated by serpins employ a proteolytic mechanism with a transition state in which the active site serine/cysteine forms a transient covalent bond with the backbone carbonyl carbon of a peptide substrate. Serpins, including AAT, have evolved to exploit this mechanism by presenting their RCL as a substrate, but prevent protease dissociation by undergoing a rapid conformational change prior to hydrolysis of the covalent bond.

It has long been recognised that, as a consequence of this substrate‐like behaviour, the sequence of the RCL is the critical determinant of the ability of a serpin to target a given protease. Accordingly, it has been possible to transfer specificity from one molecule to another by transplanting this structural element [17, 25]. It has also been observed that even when not bound, this region is primed to interact with the protease by adopting a substrate‐like conformation [21]. As the RCL sequence is optimised according to the substrate preference of the protease, typically for trypsin‐like serine proteases, the P1 residue plays a particularly important role in this recognition, but adjacent residues also contribute [25].

While the RCL represents a key interface between the two molecules, it sits adjacent to other structural elements that could feasibly represent additional sites of interaction. Several serpins exploit such exosites to refine specificity for their target proteases or to modulate the interaction in the presence of specific cofactors [15]. The direct contribution of exosites has been demonstrated for the inhibition of tPA by PAI‐1 [26] and of plasmin by the C‐terminal extension of α_2_‐antiplasmin [27]. Heparin regulation of antithrombin inhibition of the blood coagulation proteinases thrombin, fXa and fIXa, represents a prominent example of regulation by a cofactor. Heparin‐binding activates antithrombin both by allosteric effects and by acting as a bridge between the serpin to the proteinases [28]. Thrombin is also well‐recognised to expose distinct exosites that are associated with interactions with the hirudin‐like domain of the serpin heparin cofactor II, which are modulated by glycosaminoglycans [29].

The role played by AAT in protecting tissue from damage by NE is highlighted by the lung pathology that emerges in AATD when AAT plasma concentration is reduced. Despite the pathophysiological importance of the AAT‐NE interaction, currently no atomic structure exists of the encounter complex between these molecules. To address this, we used a stepwise computational approach centred around MD simulations, which provide a means to infer detailed information on dynamics in the solution state that the X‐ray crystal structures, which represent ‘snapshots’ compatible with crystal lattice formation, do not provide. Starting from the native uncomplexed AAT structures identified by cluster analysis, and with reference to the experimentally determined structure of AAT Pittsburgh with S195A trypsin, we docked NE by means of steered dynamics, followed by standard MD simulations (Fig. 2). This resulted in models of the AAT‐NE complex that proved to be stable over hundreds of nanoseconds. Despite large‐scale overall motions observed in the complex, some parts of the RCL had a significant reduction in mobility due to the constraints of binding, while other parts maintained a high level of conformational freedom. This would be expected to both favour binding, by reducing the entropic penalty associated with the transition from an RCL‐unbound to bound state, and ensure an RCL primed to insert into β‐sheet A following cleavage. Structural analyses of the MD trajectories revealed interface regions of both a polar and hydrophobic nature. In agreement with a study in which substitutions in the P3‐P3′ region of the RCL were shown to decrease the association rate and inhibitory capacity of NE by AAT [11], the models showed key interactions to involve P4‐P3′ residues in particular.

Notably, our computational analysis provides the first evidence of consistent contacts outside the RCL involved in the stabilisation of the interface between the AAT and NE molecules. A negatively charged region of AAT (residues Glu199, Asp202, Glu204) was predicted to form polar contacts with a positively charged patch on NE mediated by Arg147 (Fig. 6). Interestingly, this is in the vicinity of residues of the antithrombin exosite on the adjacent β‐strand 3C critical to the promotion of rapid inhibition of factors Xa and IXa [28]. The mutagenesis experiments reported here revealed that substitution of these negatively charged residues with alanine or serine, or the introduction of a repulsive positive charge at position 202, made no or a minimal change in the inhibitory capacity of AAT but reduced the rate of interaction with NE by 30–50% (Fig. 9). The reduction of the rate of interaction of wild‐type AAT with NE in the presence of increased salt, and the lesser or absent effect seen with the mutants, is consistent with this effect arising from electrostatic interactions that are no longer formed by the latter molecules. This supports the hypothesis that this region represents an exosite that contributes to a highly optimised, charge‐based intermolecular interaction. Long‐range electrostatic interactions between this acidic region of AAT and positively charged areas of NE could also favour the approach of the two molecules, with a possible effect of electrostatic steering. However, our data are insufficient for its quantitative estimate, and it is uncertain how it could affect the reciprocal orientation, due to the extensive presence of positive charges on the surface of NE (Fig. 6). In agreement with a more specific role of the exosite in the AAT‐NE interface, within the MD simulations, a correlation was found between the formation of intermolecular contacts by the exosite and the adoption of a geometry between the P1 (Met358) residue and the protease catalytic triad (Fig. 7) compatible with the RCL cleavage. Kinetics that monitor the progressive inhibition of the protease over time primarily report the effect of productive interactions resulting in enzyme inhibition; this in turn requires proper alignment of the catalytic residues with the target peptide bond. Indeed, the experimental data presented here show that the rate of association is reduced when the exosite residues are perturbed.

The identification of this exosite extends the basis for AAT recognition of NE to a separate structural element and thereby reveals a more extensive surface area for the interaction than previously known. This has translational implications. With regards to AATD, natural mutations occurring at this exosite are unlikely to perturb protein folding, as suggested by high yields of soluble monomeric form of the recombinant AAT variants produced in bacteria. However, amino acid substitutions affecting the exosite may result in AAT variants with a reduced inhibitory function. Of note, two variations affecting this site (D202Y and E204K) are currently annotated in the gnomAD database of population SERPINA1 variants. It has been observed that in contrast to most missense mutations associated with AATD, which lead to protein misfolding and polymerisation in the endoplasmic reticulum of hepatocytes [30, 31, 32, 33], variants can exhibit normal folding and secretion by cells [11] while being functionally compromised. As a consequence, most clinical pipelines would fail to identify a carrier of these type‐2 dysfunctional variants.

It is interesting to note that the positively charged loop of NE that is predicted to interact with the novel AAT exosite has previously been proposed to bind to anionic molecules including NETs DNA, heparin and heparan‐sulphates [34, 35]. This would suggest a reduced ability of AAT to form a complex with ligand‐bound NE through charge‐mediated and steric effects, and heparin has indeed been found to decrease their rate of association [36]. Interestingly, Arg147 and the adjacent Arg149 have been proposed as a possible binding site of heparin‐mimetics, a class of noncompetitive NE inhibitors that are currently being developed for potential clinical application [37, 38].

Here we used an interdisciplinary approach, combining computational and biochemical analyses, to define the AAT‐NE interface, revealing the contribution of an exosite with complementary electrostatic character on each molecule. Previous observations of binding of negatively charged biomolecules by NE, and the resulting decrease in the rate of interaction with AAT, raise the possibility that this interaction may be modulated by extrinsic factors in the local environment. These findings have implications relevant to the pathophysiology of AATD and other lung diseases with NE involvement.

## Materials and methods

### Determination of initial structures

The initial structure for the simulations of AAT was taken from PDB entry 1QLP [5]. For the AAT‐NE complex, we used as a reference structure 1OPH, which represents the complex of the M358R variant of AAT with bovine trypsin carrying the S195A substitution. The crystal structure of NE was taken from PDB entry 3Q76 [20]. In this work, NE residues are numbered as in 3Q76, which adopted the conventional numbering scheme based on NE alignment to bovine chymotrypsin A, and not that of the NE sequence in the UniProtKB database (ID P08246).

Mutations were introduced into the AAT structure (PDB 1QLP) or the complex derived from an AAT‐NE trajectory by mutagenesis in PyMOL (Schrödinger, LLC, New York, NY, USA).

### Standard molecular dynamics

The software package GROMACS [39] was used for MD simulations, with force field amber99‐sb. The Coulomb interactions were treated by the PME method and the Lennard‐Jones potential was employed for short‐range interactions, with a cut‐off of 10 Å. After addition of hydrogens, where necessary, to the initial structure, solvation with TIP3P water was applied in a triclinic simulation box with boundaries at 10 Å minimum distance from the solute. By addition of Na^+^ and Cl^−^ ions, an overall neutral charge and a salt concentration of 150 mm were obtained. Energy was minimised and two 100 ps equilibration runs, one at constant volume and one at constant pressure, were performed with restraints on heavy atoms, before proceeding with unrestrained simulations at constant temperature and pressure. The velocity rescaling method was used to keep temperature constant (at 310 K in all cases) with a characteristic time of 0.1 ps. A constant pressure was enforced by the Parrinello–Rahman algorithm with a time constant of 1 ps and a compressibility of 4.5∙10^−5^ bar^−1^.

### Essential dynamics

The essential dynamics algorithm available in the gromacs package [39] as ‘acceptance linear expansion’ was applied to the collective coordinate corresponding to the relative motion of the two centres of mass. To this end, a suitable vector in the configuration space of the AAT‐NE complex was calculated and was regarded as an eigenvector in the essential dynamics (.edi) input file.

### Trajectory postprocessing

Several structural analyses (such as atomic distances, solvent accessible surface area, hydrogen bonds) and the principal component analysis (PCA) were performed by means of tools available in the gromacs package [39]. Other tools, concerning cluster analysis, generation of input structures and data for essential dynamics, evaluation of hydrophobic contacts and calculation of other observables derived from PCA, were developed in‐house.

Electrostatic potential calculations were performed by means of the APBS software [40]. Software packages vmd [41] and PyMOL (Schrödinger, LLC) were used for structure manipulation and visual representation.

Interaction energy calculations by means of the MMGB/SA method were performed by our own computer program. The generalised Born approximation for the calculation of the polar solvation contribution to the binding energy was implemented according to [24]. We recall here that, in this model, the solvation energy of a molecule is given by

$$\Delta E=-\frac{1}{2}\sum \frac{q_{i}q_{j}}{f_{\mathrm{GB}}\left(r_{\mathrm{ij}},R_{i},R_{j}\right)}$$

with

$$f_{\mathrm{GB}}\left(r_{\mathrm{ij}},R_{i},R_{j}\right)={\left(r_{\mathrm{ij}}^{2}+R_{i}R_{j}\exp\left(-r_{\mathrm{ij}}^{2}/4R_{i}R_{j}\right)\right)}^{\frac{1}{2}}$$

$$\begin{array}{l} R_{i}^{-1}=\rho_{i}^{-1}-\rho_{i}^{-1}\tan\;h\left(\alpha \Psi -{\beta \Psi }^{2}+{\gamma \Psi }^{3}\right),\Psi =I\rho_{i}^{\prime },\\ I=\frac{1}{4\pi }\int \frac{1}{r^{4}}d^{3}r \end{array}$$

where *r*_*ij*_ is the distance between atoms *i* and *j*, *r*_*i*_ is the atomic radii, *ρr'*_*i*_ = *rρ*_*i*_ − *δ* are the atomic radii reduced by an offset and the integration region for atom *i* is the interior of the molecule, excluding the sphere of atom *i*. The following parameters were used: α = 0.8, β = 0.0, γ = 2.91, δ = 0.09 Å. Integrals *I* were calculated by means of a Monte Carlo procedure. Atomic radii were taken from GROMACS parameters [39].

### Preparation of expression vectors by site‐directed mutagenesis

The expression vector pQE31 encoding human AAT cDNA with an MRSHHHHHH tag at the N terminus was described previously [42]. Vectors encoding the AAT variants were obtained by the QuikChange II site‐directed mutagenesis kit (Agilent, Milan, Italy), according to the manufacturer's instructions, using the following oligonucleotide primers and their reverse complements: 5′‐GAGAGACCCTTTGCAGTCAAGGCCACCGCGGAAGAGGACTTC (3×Ala); 5′‐CTTCTTTAAAGGCAAATGGGAGAGACCCTTTTCAGTCAAGAGCACCTCGGAAGAGGACTTCCAC (3×Ser);5′‐CCCTTTGAAGTCAAGCGCACCGAGGAAGAG (D202R). The successful mutations were confirmed by DNA sequencing.

### Bacterial expression and purification of recombinant AAT

The recombinant AAT variants were expressed in the *E. coli* XL1‐blue strain (Agilent). Bacteria were grown in terrific broth with 100 μg·mL^−1^ ampicillin and 1 mm isopropyl‐b‐d‐thiogalactopyranoside (IPTG, Merck, Darmstadt, Germany) for 24–36 h at 24 °C. The cells were collected by centrifugation, resuspended in chilled PBS/0.02% sodium azide, treated with lysozyme (Merck), frozen and thawed, and further disrupted by sonication. Cell debris was removed by centrifugation at 16 000 ***g*** for 20 min, and the supernatant was filtered with glass fibre filter. The AAT variants were purified using the ÄKTA Pure™ chromatography system (Cytiva, Marlborough, MA, USA) with a 5 mL HisTrap™ FF crude column in 20 mm Tris pH 8.0/0.02% sodium azide and elution with an imidazole gradient (0–250 mm), and by ion exchange using a 5 mL HiTrap™ Q Sepharose column and elution in a gradient of NaCl (0–1 m) in 20 mm Tris pH 8.0/0.02% sodium azide. The fractions with the highest concentration of protein were pooled, quantified spectrophotometrically using an extinction coefficient ε_1%_ of 5.8 and stored in aliquots at −80 °C.

### PAGE and western blot analysis of recombinant AAT variants

Recombinant AAT variants were analysed either by reducing SDS/PAGE, using 4–12% w/v acrylamide Bolt™ Bis‐Tris Mini Protein Gels (Thermo Fisher Scientific, Waltham, MA, USA) in Bolt™ MES running buffer (Thermo Fisher Scientific), or by nondenaturing‐PAGE, using Novex™ Value™ 4–12% w/v acrylamide Tris‐Glycine Mini Protein Gel (Thermo Fisher Scientific) resolved in 0.1 m Tris/HCl pH 8.0, as described previously [43]. Gels were stained by 0.1% w/v Coomassie Blue.

### Detection of AAT‐NE complexes by western blot

Recombinant AAT variants (8 nm) were incubated with increasing concentrations (0, 4, 8, 12 nm) of purified human NE (Athens Research & Technology, Athens, GA, USA) in PBS/0.05% NP‐40/0.5 m NaCl for 1 h at room temperature. Proteins were resolved by SDS/PAGE in reducing conditions on 4–12% Bolt™ Bis‐Tris Mini Protein Gels (Thermo Fisher Scientific) and blotted to Hybond‐P 0.45 PVDF membranes (Cytiva). For western blot analysis, membranes were incubated with defatted milk diluted in TBS containing 0.05% w/v Tween‐20 (TBS‐T) for 1 h at room temperature. AAT was detected by incubating membranes with 2 μg·mL^−1^ rabbit polyclonal antibody anti‐AAT (A001202; Dako, Milan, Italy), overnight at room temperature, and then by incubating with 100 ng·mL^−1^ HRP‐conjugated donkey anti‐rabbit IgG (NA9340; Cytiva) in TBS‐T, for 1 h at room temperature. Signals were developed with SuperSignal™ West Pico PLUS Chemiluminescent Substrate (Thermo Fisher Scientific) and captured by the Odyssey Fc imager (LI‐COR Biosciences, Bad Homburg, Germany).

### Differential scanning fluorimetry

To assess the thermal stability of AAT and mutants, 20 μL samples comprising protein (0.1 mg·mL^−1^ final concentration) and SYPRO Orange dye (5× final concentration) (Thermo Fisher Scientific) in PBS, were heated from 25 to 95 °C at a rate of 0.5 °C/30 s and fluorescence monitored using a RealPlex 4 thermal cycler (Eppendorf). The midpoint of denaturation, *T*_*m*_, was determined from the maximum of the first derivative of the curve using a 7‐point window of spline‐fitted data in Prism 10 (GraphPad Inc., Boston, MA, USA).

### Circular dichroism spectroscopy

To establish the secondary structure profile of AAT and mutants, spectra were collected on a Chirascan V100 circular dichroism spectrometer (Applied Photophysics, Leatherhead, UK) in a Hellma Macro quartz glass 1 mm cuvette using protein samples extensively dialysed into CD buffer (50 mm sodium phosphate pH 7.4) to remove any chloride ions. Three far‐UV scans, from 260 to 180 nm, were collected per sample and averaged.

### Stoichiometry of inhibition assay

To determine the SI of NE inhibition, dilutions of AAT were incubated with a fixed concentration of human NE (5 nm) in PBS/0.05% v/v NP40/0.5 mm NaCl (AAT/NE ratio: 1.5, 1, 0.8, 0.6, 0.4, 0.2, 0.1, 0). After 1 h incubation, the activity of residual uncomplexed NE was measured by the turnover of the chromogenic substrate methoxy‐Suc‐Ala‐Ala‐Pro‐Val‐p‐nitroanilide (Merck), by reading the 410 nm absorbance every minute for 30 min at 25 °C, utilising the Ensight® multimode plate reader (PerkinElmer). Rates of turnover were determined for each AAT/NE ratio by fitting the absorbance values to a linear regression and calculating their slopes. Those slopes were then plotted as a function of the AAT/NE ratios and fitted to a linear regression, whose x‐intercept yielded the SI.

### Rate constants of NE inhibition

To determine the second‐order association rate constant, AAT variants were mixed with purified human NE (Athens Research & Technology) and its fluorogenic substrate MeO‐Suc‐Ala‐Ala‐Pro‐Val‐AMC (Cayman) in PBS/0.05% v/v NP40 or PBS/0.05% v/v NP40/0.5 m NaCl buffer, to a final volume of 200 μL per well, in black nonbinding 96‐well microplates (655 076; Greiner Bio‐One, Milan, Italy) [11, 44]. To obtain pseudo‐first order conditions we used 7.5‐, 10‐ and 12.5‐fold molar excess of active AAT (adjusted by the SI value determined for each variant) over NE (0.25 nm). The NE substrate was added with an excess of 2.5 times the Michaelis–Menten constant of NE (*K*_M_ = 293 μm). The fluorescent signal resulting from substrate hydrolysis was monitored by excitation at 367 nm and emission at 460 nm wavelengths, every 5 s for 30 min at 25 °C using the EnSight® multimode plate reader (PerkinElmer). For each AAT variant, the *k*_obs_ was obtained by plotting the fluorescent signal over time and fitting the data to Eq. $\left(1\right)$; then *k*_inh_—the apparent second‐order rate constant of inhibition—was calculated according to Eq. $\left(2\right)$ [45].

(1) $$F=V_{s}t+\frac{\left(V_{i}-V_{s}\right)\left(1-e^{-k_{\mathrm{obs}}t}\right)}{k_{\mathrm{obs}}}+\text{baseline}$$

*F*, fluorescence units; *V*_*i*_, initial rate; *V*_*s*_, final rate; *t*, time; *k*_*obs*_, observed rate constant.

(2) $$k_{\mathrm{inh}}=k_{\mathrm{obs}}\cdot \frac{\left(1+\frac{\left[S\right]}{K_{M}}\right)}{\left[\mathrm{AAT}\right]}$$

*k*_*inh*_, inhibition rate constant; *k*_*obs*_, observed rate constant; [S], molar concentration of substrate; *K*_*M*_, Michaelis–Menten constant of NE substrate hydrolysis; [AAT], molar concentration of AAT.

*k*_*ass*_ was calculated as:

(3) $$k_{\mathrm{ass}}=k_{\mathrm{inh}}\cdot \mathrm{SI}$$

## Conflict of interest

The authors declare no conflict of interest.

## Author contributions

RG: data curation, formal analysis, investigation, methodology, and software. MB: investigation, methodology, formal analysis, and visualisation. AD: investigation and methodology. CNP: Investigation and formal analysis. RR: investigation and methodology. DAL: funding acquisition, resources and writing—review and editing. JAI: conceptualisation, funding acquisition, methodology, formal analysis, writing original draft, writing—review and editing. AF: conceptualisation, formal analysis, funding acquisition, methodology, writing original draft, writing—review and editing. FG: conceptualisation, methodology, resources, software, writing original draft, writing—review and editing.

### Peer review

The peer review history for this article is available at https://www.webofscience.com/api/gateway/wos/peer‐review/10.1111/febs.17387.

## Supporting information


**Figure S1.** Principal component analysis. Collective motion corresponding to the first principal component obtained from the MD simulations of the AAT‐NE complex. Animation produced through VMD [41].

**Figure S2.** Principal component analysis. Collective motion corresponding to the second principal component obtained from the MD simulations of the AAT‐NE complex. Animation produced through VMD [41].

**Figure S3.** Principal component analysis. Collective motion corresponding to the third principal component obtained from the MD simulations of the AAT‐NE complex. Animation produced through VMD [41].

**Video S1.** Essential dynamics. Excerpt from one ED simulation showing the approach of NE to AAT. Animation produced through VMD [41].

**Video S2.** Essential dynamics detail. Excerpt from one ED simulation showing the approach of NE to AAT with a focus on the interface region. Animation produced through VMD [41].

## Data Availability

MD simulation trajectories and some related files are available on the Zenodo data repository, at the following address: https://doi.org/10.5281/zenodo.11299542.
