# Supplementary figures and images for: Identification of an exosite at the neutrophil elastase/alpha‐1‐antitrypsin interface
Source: FEBS J. 2025 Jan 8;292(8):1887–903. doi: 10.1111/febs.17387 (PMC12001179; doi:10.1111/febs.17387)

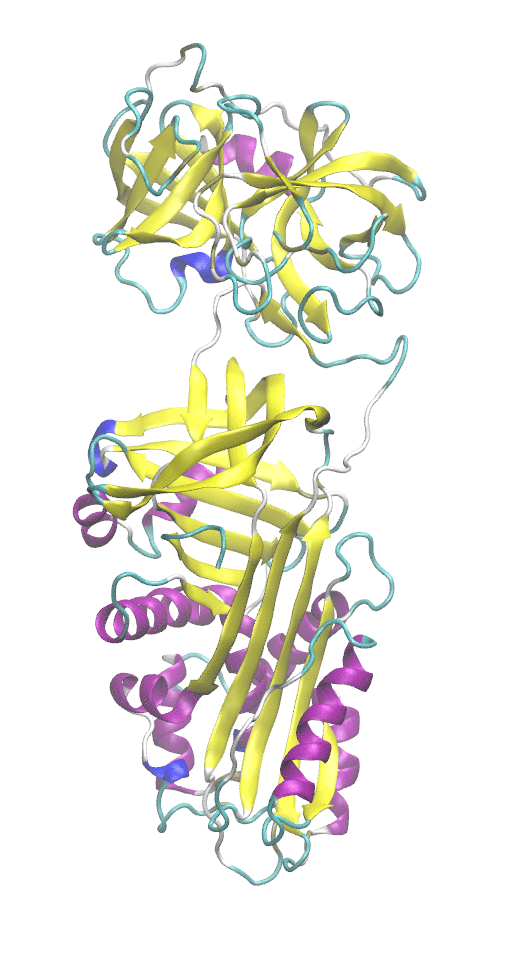

Supplement: Supplementary file 1 — Figure S1. Principal component analysis. Collective motion corresponding to the first principal component obtained from the MD simulations of the AAT‐NE complex. Animation produced through VMD [41]. [file FEBS-292-1887-s004.gif]

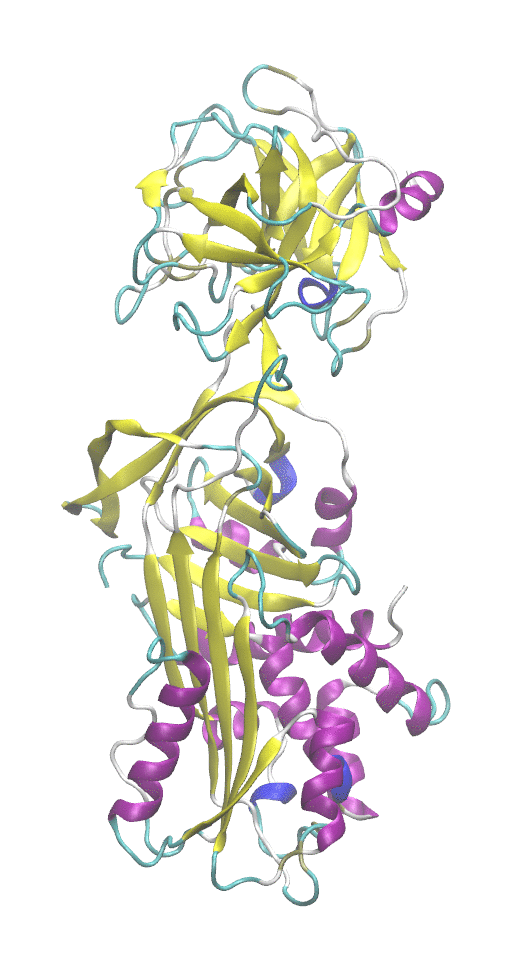

Supplement: Supplementary file 2 — Figure S2. Principal component analysis. Collective motion corresponding to the second principal component obtained from the MD simulations of the AAT‐NE complex. Animation produced through VMD [41]. [file FEBS-292-1887-s005.gif]

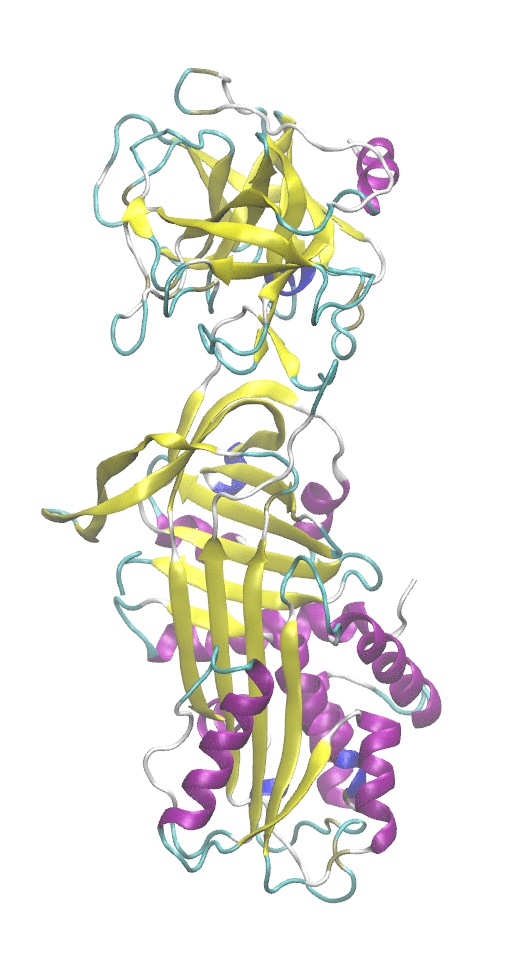

Supplement: Supplementary file 3 — Figure S3. Principal component analysis. Collective motion corresponding to the third principal component obtained from the MD simulations of the AAT‐NE complex. Animation produced through VMD [41]. [file FEBS-292-1887-s002.gif]
